# *Pectobacterium brasiliense 1692* Chemotactic Responses and the Role of Methyl-Accepting Chemotactic Proteins in Ecological Fitness

**DOI:** 10.3389/fpls.2021.650894

**Published:** 2021-04-22

**Authors:** Collins Kipngetich Tanui, Divine Yutefar Shyntum, Precious K. Sedibane, Daniel Bellieny-Rabelo, Lucy N. Moleleki

**Affiliations:** ^1^Department of Microbiology and Plant Pathology, University of Pretoria, Pretoria, South Africa; ^2^Forestry and Agricultural Biotechnology Institute, University of Pretoria, Pretoria, South Africa

**Keywords:** chemotaxis, soft rot, blackleg disease, *Pectobacterium brasiliense*, ecological fitness, virulence, methyl accepting chemotaxis proteins

## Abstract

To adapt to changing environmental niches, bacteria require taxis, a movement toward or away from a stimulus (ligand). Chemotaxis has been studied in some members of the Soft Rot Pectobacteriaceae (SRP), particularly members of the genus *Dickeya*. On the contrary, there are fewer studies on this topic for the other genus in the SRP group, namely *Pectobacterium*. This study evaluated chemotactic responses in *Pectobacterium brasiliense* (*Pb 1692*) to various ligands. A total of 34 methyl-accepting chemotactic proteins (MCPs) were identified in the *Pb 1692* genome and the domain architectures of these MCPs were determined. Four *Pb 1692* MCPs previously shown to be differentially expressed during potato tuber infection were selected for further functional characterization. Toward this end, *Pb 1692* mutant strains each lacking either *AED-0001492*, *AED-0003671*, *AED-0000304*, or *AED-0000744* were generated. Two of these mutants (*AED-0001492* and *AED-0003671*), were attenuated in their ability to grow and respond to citrate and are thus referred to as *MCP_*cit2*_ and MCP_*cit1*_*, respectively, while the other two, *AED-0000304* (*MCP*_*xyl*_) and *AED-0000744* (*MCP*_*asp*_), were affected in their ability to respond to xylose and aspartate, respectively. Trans-complementation of the mutant strains restored swimming motility in the presence of respective ligands. The four MCP mutants were not affected in virulence but were significantly attenuated in their ability to attach to potato leaves suggesting that ecological fitness is an important contribution of these MCPs toward *Pb 1692* biology.

## Introduction

Bacteria employ a myriad of mechanisms to efficiently adapt to changing environmental conditions (Armitage, 1992; Blair, 1995). Such pieces of machinery include one-component systems (OCS), two-component systems (TCS), and chemoreceptor-based signaling, also known as chemotaxis (Matilla and Krell, 2017). Chemotaxis is an important process involving several host-pathogen interactions, and can ultimately determine the outcome of infection (Armitage, 1992; O’Toole et al., 1996; Antúnez-Lamas et al., 2009a). Chemotaxis and motility are critical during the early stages of infection when bacteria search for entry sites to penetrate the host apoplast (Stock and Baker, 2009; Reverchon and Nasser, 2013). The attracted bacteria swim toward a wounded site to which they attach and subsequently enter the host apoplastic environment (Antúnez-Lamas et al., 2009a).

In bacteria, methyl-accepting chemotaxis proteins (MCPs) or chemoreceptors are membrane-bound receptors that sense external stimuli and respond through a signal transduction pathway consisting of CheABRWYZ proteins (Charkowski et al., 2012). The molecular mechanism of taxis has been extensively studied in *Escherichia coli* and this bacterium has been shown to have five MCPs (Stock and Baker, 2009). However, unlike *E. coli*, many plant pathogens are predicted to encode more than 30 MCP receptors; with different *Pectobacterium* spp. encoding between 30 and 39 taxis receptors (Glasner et al., 2008).

Chemoreceptors respond to different ligands such as sugars, amino acids, and organic acids (Lacal et al., 2010). The ligand-binding domains (LBDs) of MCPs can either be located in the periplasmic or cytoplasmic space (Porter et al., 2011). The most abundant LBDs belong to three superfamilies, namely the 4-helix bundle (4HB), CACHE, and the PAS domain (the PAS domain is typically cytosolic in location) (Ortega et al., 2017). Following the perception of this signal, the LBD induces a conformational change that triggers the chemosensory signaling cascade leading ultimately to chemotaxis (Porter et al., 2011). This conformational change leads to alterations in CheA autokinase activity. CheA is a cytoplasmic histidine autokinase protein that interacts with MCPs through CheW, a coupling protein (Falke and Hazelbauer, 2001). The phosphorylated CheA then transfers its phosphoryl group to a diffusible cytoplasmic response regulator CheY (Falke and Hazelbauer, 2001). The phosphorylated CheY rotates the flagellar either clockwise or anti-clockwise by binding to the motor region of the flagellar, resulting in swimming motility away from repellents or toward favorable conditions (Falke and Hazelbauer, 2001). *Pectobacterium brasiliense*, a member of the soft rot *Pectobacteriaceae* (SRP), is a concern to potato growers worldwide (Duarte et al., 2004; van der Merwe et al., 2010; Panda et al., 2012; Onkendi et al., 2014). Unlike *Dickeya dadantii*, another member of the SRP, responses of *P. brasiliense* to various ligands are not well established (Rio-Alvarez et al., 2012). As a consequence, the roles of *Pb 1692* chemoreceptors in response to various substrates during the infection process are still poorly understood. A previous report from our laboratory found several chemoreceptors differentially expressed in *Pb 1692* during potato tuber infection (Bellieny-Rabelo et al., 2019). Based on these observations, four differentially expressed chemoreceptors were selected for further characterization. In this study, we evaluated the response of *Pb 1692* wild-type and four MCP mutant strains to 20 sugars, amino, and organic acids. Furthermore, the four MCP mutant strains were assessed for their ability to confer fitness advantage during *in vitro* growth and during plant colonization relative to the wild-type strain.

## Results

### Identification of Methyl-Accepting Genes in *Pb 1692*

**FIGURE 1:**
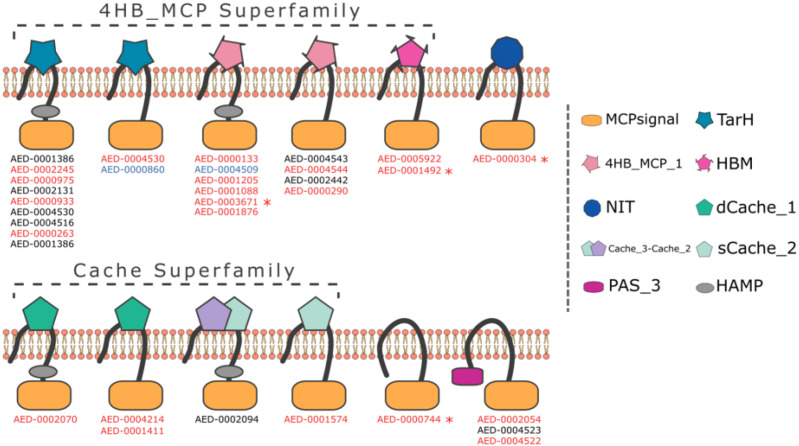
Representation of domain organization of all 34 MCP in *Pb 1692* strain. The organization containing both MCP and HAMP domains is the most common in *Pb 1692* strain. All predicted domain structures were made through HMMER software with the support of the Pfam-A database. Transmembrane regions were predicted by the Phobius algorithm. The genes highlighted: (1) black was not upregulated, (2) blue was down-regulated, and (3) red was up-regulated during *in-planta* infection. Furthermore, genes with “*” indicated those mutated and characterized.

Using the ASAP database^1^ we screened the *Pb 1692* genome for the presence of methyl-accepting proteins (MCPs). The similarity-based approach (BLASTP) detected 34 taxis receptor proteins containing a methyl-accepting protein domain in the *Pb 1692* genome (Supplementary Table 1). Domain architecture analyses indicated that all 34 chemoreceptor proteins harbor a cytosolic MCP domain and a few (5/34) have an additional HAMP (histidine kinases, adenylyl cyclases, MCPs, and phosphatases) linker domain. However, at this stage we cannot rule out the possibility that receptors identified here as being without a HAMP domain do indeed have one. The most abundant domains were those belonging to the 4HB and CACHE superfamilies. Other domain organizations include TarH, NIT, PAS, and the helical bimodular (HBM) domain (Figure 1 and Supplementary Table 1).

### Generation of *Pb 1692* MCP Mutant Strains

Four MCPs (AED-0001492, AED-0003671, AED-0000304, and AED-0000744) representing different architectural classes were selected for further functional characterization. AED-0000304 has a NIT ligand-binding domain while AED-0000744 is cytosolic. Both AED-0001492 and AED-0003671 belong to the 4HB superfamily. An alignment of AED 00003671 and AED 0001492 showed a 61% similarity and both these proteins present transmembrane regions in similar locations (Supplementary Figures 1, 2). All four selected MCP genes were differentially expressed in *Pb 1692* at 24 h post-infection (hpi) in potato tubers (Bellieny-Rabelo et al., 2019), suggesting they might play an important role at this stage of infection. To investigate the role of these four MCPs in *Pb 1692* concerning ecological fitness as well as virulence, we generated isogenic mutant strains each lacking one of these genes (Supplementary Figure 3). The integrity of the mutant strains was confirmed by a Southern blot, PCR, and sequencing (Supplementary Figure 3, and results not shown). The mutant strains were successfully complemented by expressing the wild-type gene of interest extra-chromosomally (using a plasmid) in the mutated strains.

### Chemotaxis Response of *Pb 1692* Wild-Type and Selected Mutants to Different Ligands

The *Pb 1692* wild-type was screened for the ability to grow and respond to different chemo-attractants consisting of 20 amino acids (aa), sugars, and organic acids supplemented in M9 minimal media (Table 1). Responses were categorized into three groups namely; highly (2 cm and above), moderately (1.0–2.0 cm), and least (0.1–1.0 cm) responsive based on the *Pb 1692* wild-type motility in M9 minimal soft-agar plates. The results indicated that *Pb 1692* wild-type was highly responsive to some sugars and several amino acids listed in Table 1. We observed that *Pb 1692* wild-type was moderately responsive to most amino acids as well as one sugar, sucrose. On the contrary, *Pb 1692* wild-type was least responsive to, or in some cases unable to grow in the presence of the following; maleic acid, maltose, mannose, and cysteine (Table 1).

**TABLE 1:** Halo diameter (cm) in motility plate supplemented with 1 mM chemical substance.

Growth conditions	Halo diameter (cm) in motility plate supplemented with 1 mM chemical substance								
	*Pb1692*	Δ*MCPcit1*	*c*Δ*MCPcit1*	Δ*MCcit2*	*c*Δ*MCPcit2*	Δ*MCPasp*	*c*Δ*MCPasp*	Δ*MCPxyl*	*c*Δ*MCPxyl*
**Highly responsive**									
M9 + Glucose	2.2	2.1	2.1	2.2	2.1	2.2	2.2	2.1	2.2
M9 + Citric acid	2.3	0.2	2.2	0.3	2.2	2.3	2.3	2.2	2.3
M9 + Aspartate	2.4	2.3	2.2	2.3	2.4	0.1	2.4	2.3	2.4
M9 + Aspartic acid	2.5	2.4	2.4	2.5	2.5	0.1	2.4	2.5	2.5
M9 + Xylose	2.1	2.0	2.0	2.1	2.1	2.1	2.0	0.1	2.1
M9 + Fructose	2.3	2.2	2.2	2.1	2.2	2.1	2.1	2.2	2.2
M9 + Jasmonic acid	2.4	2.3	2.4	2.4	2.3	2.4	2.3	2.4	2.4
M9 + Glutamic acid	2.3	2.3	2.2	2.2	2.3	2.3	2.3	2.2	2.3
M9 + Ribose	2.5	2.4	2.5	2.4	2.4	2.5	2.4	2.5	2.5
M9 + Arginine	2.0	1.9	2.1	2.0	1.9	2.0	2.0	2.1	1.9
M9 + Valine	2.2	2.1	2.2	2.0	2.1	1.9	2.0	2.1	2.0
M9 + Glutamine	2.3	2.2	2.2	2.3	2.3	2.2	2.3	2.2	2.3
M9 + Methionine	2.3	2.3	2.2	2.3	2.3	2.3	2.3	2.3	2.2
**Moderately responsive**									
M9 + Sucrose	1.2	1.1	1.1	1.2	1.1	1.0	1.1	1.2	1.1
M9 + Asparagine	1.5	1.5	1.4	1.5	1.5	1.4	1.4	1.5	1.6
M9 + Alanine	1.4	1.3	1.3	1.4	1.3	1.3	1.3	1.4	1.5
M9 + Histidine	1.1	1.0	1.0	1.1	1.1	1.1	1.2	1.1	1.2
M9 + Phenylalanine	1.9	1.9	1.8	1.8	1.8	1.9	1.9	1.8	1.8
M9 + Serine	1.7	1.7	1.8	1.7	1.8	1.8	1.7	1.7	1.7
M9 + Leucine	1.2	1.1	1.2	1.1	1.2	1.2	1.2	1.1	1.1
**Least responsive**									
M9 + Maleic acid	0.1	0.1	0.1	0.1	0.1	0.1	0.1	0.1	0.1
M9 + Maltose	0.4	0.4	0.3	0.3	0.4	0.4	0.3	0.3	0.4
M9 + Mannose	0.1	0.1	0.1	0.1	0.1	0.1	0.1	0.1	0.1
M9 + Cysteine	0.2	0.2	0.2	0.2	0.2	0.2	0.2	0.2	0.2

**FIGURE 2:**
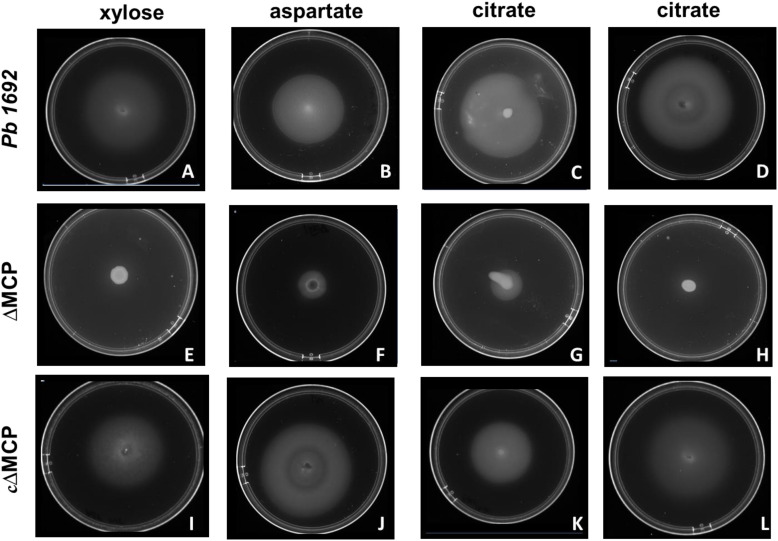
Swimming motility assay. *Pb* 1692 wild-type was spotted on minimal media supplemented with 1 mM of xylose **(A)**, aspartate **(B)**, and citrate **(C,D)**. Similarly, mutant strains lacking *AED-0000304* (*Pb1692*Δ*MCP*_*xyl*_), *AED-0000744* (*Pb1692*Δ*MCP*_*asp*_), *AED-0003671* (*Pb1692*Δ*MCP*_*cit1*_), and *AED-0001492* (*Pb1692*Δ*MCP*_*cit2*_) were spotted on minimal media supplemented with 1 mM of xylose **(E)**, aspartate **(F)** and citrate **(G,H)**, respectively. Complemented strains *Pb1692*Δ*MCP*_*xyl*__–_p*xyl Pb1692*Δ*MCP*_*asp*__–_p*asp*, *Pb1692*Δ*MCP*_*cit1*__–_p*cit1*, and *Pb1692*Δ*MCP*_*cit2*__–_p*cit2*, were spotted on minimal media supplemented with 1 mM of aspartate **(I)**, xylose **(J)** citrate **(K,L)**, respectively.

To determine the specific ligands associated with each of the four *Pb 1692* MCP mutant strains, we screened for chemotactic responses of the four *Pb 1692* MCP mutants, wild-type and complemented mutant strains toward the 20 chemo-attractants (Table 1 and Figures 2A–L). The results demonstrated that *AED-0000304* and *AED-0000744* mutants were least attracted to xylose and aspartate, respectively (Figures 2E,F) hence these were denoted *Pb1692*Δ*MCP*_*xyl*_ and *Pb1692*Δ*MCP*_*asp*_, respectively. On the other hand, both *Pb 1692 AED-0001492* and *AED-0003671* mutants were least attracted to citrate compared to other *Pb* strains (Figures 2G,H) hence these were denoted *Pb1692*Δ*MCP_*cit*__2_* and *Pb1692*Δ*MCP_*cit*__1_*, respectively. Trans-expression of (*MCP*_*xyl*_), (*MCP_*asp*_)*, and *MCP_*cit*__2_* and *MCP_*cit*__1_* genes in the corresponding mutant strains restored chemotaxis of the complemented strains to wild-type levels (Figures 2I–L). Given that MCPs have been associated with the ability of bacteria to optimally utilize different amino acids and carbon sources, we compared the growth rate of *Pb 1692* wild-type and its respective MCP mutant strains in different amino acids and carbon sources. Our findings were in agreement with the results obtained from carbon utilization assays where utilization of sole carbon source was determined (Table 1). The response toward glucose, fructose, sucrose, mannose, ribose, glutamate, valine, alanine, serine, methionine, and asparagine as sole sources of carbon was also found to be similar between the different *Pb 1692* MCP mutant strains and the wild-type (Table 1). It was also observable that, *Pb 1692* wild-type and the mutant strains generated in this study were least attracted to or unable to grow in the presence of mannose, maltose, maleic acid, and cysteine (Table 1).

### MCP Mutant Strains Are Attenuated in Attachment but Not Virulence

**FIGURE 3:**
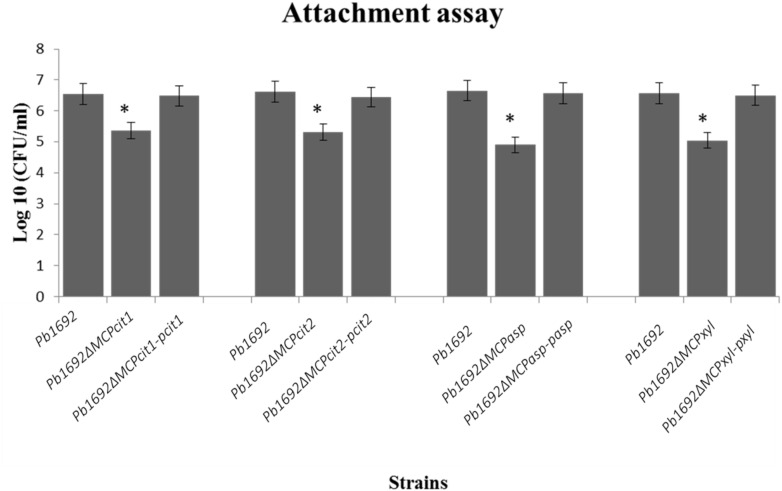
MCP mutants were attenuated in their ability to attach to potato leaves. Determination of CFU/ml from potato leaves after 2 h incubation with 10 μl of each mutant strain (OD_600_ equivalent to 1.0) in M9 media. Three independent experiments were performed each with three technical replicates with a corresponding standard error of the mean. Statistically significant differences (*p* < 0.05) between mutants, wild-type, and complemented strains are shown by an asterisk.

Some bacteria use different MCPs to sense and move toward different plant signals, for example, jasmonic acid produced by wounded plants (Río-Álvarez et al., 2015). Here, we wanted to determine the role played by these four *Pb 1692* MCPs in the attachment to potato leaves. The CFU/ml count indicated that mutant strains were significantly reduced in their ability to attach and colonize potato leaves when compared to *Pb 1692* wild-type strain (Figure 3). Conversely, *in vitro* growth and virulence in potato tubers indicated that the mutant strains and *Pb 1692* wild-type strain had no significant difference (Supplementary Figures 4A,B). Together, these findings demonstrate that *in planta* and *in vitro* growth as well as virulence on potato tubers are not affected by deletion of these selected MCP-encoding genes in *Pb 1692*.

## Discussion

Chemotaxis plays an important role in phytopathogenic bacteria host colonization, infection, and disease development. In our previous work (Bellieny-Rabelo et al., 2019), we observed that the master regulator for flagellar biogenesis (*FlhDC*) together with other genes encoding flagellar biosynthesis (*Flg and Fli*), chemotactic signaling cascade (*CheADVWYZ*), and various MCPs were mostly upregulated at 24 hpi during *Pb 1692* infection of potato tubers. This indicated that the bacteria are potentially responding to some host-derived environmental cues or ligands leading to effective host colonization. However, to date, responses of *Pb 1692* to different types of ligands have not yet been documented. Furthermore, unlike *Dickeya*, chemotaxis is less studied for most *Pectobacterium* spp. (Antúnez-Lamas et al.,2009a,b; Río-Álvarez et al., 2015; Velando et al., 2020). Toward this end, our first aim was to determine *Pb 1692* responses to various ligands. Our results show that citrate, xylose, glucose, fructose, jasmonic acid, and a number of amino acids are amongst those ligands to which *Pb 1692* is highly attracted to. This can be expected since potato tubers have high concentrations of sugars such as fructose and glucose, organic acids such as malic and citric acid as well as various amino acids, albeit in varying concentrations per cultivar (Dobson et al., 2008; Uri et al., 2014). Also, xylose is a monomer of xylan released by plant cells upon damage (Beliën et al., 2006). Interestingly, the gene that drives the strong response to xylose (*MCP*_*xyl*_) was recently reported as part of a SlyA regulatory network in *Pb 1692* (Bellieny-Rabelo et al., 2020). More specifically, the SlyA regulon at 12 hpi on potato tubers is enriched with genes associated with carbohydrate metabolism, and it also includes the *MCP*_*xyl*_ gene. Hence, the results presented here are consistent with these findings and may point to a yet larger carbohydrate-responsive network under the regulation of SlyA than previously thought, one that comprises xylose as one of its triggers.

Given that *D. dadantii* has been shown to swim toward jasmonic acid, it is also not surprising that jasmonic acid is one of those metabolites that *Pb 1692* was highly attracted to Hugouvieux-Cotte-Pattat and Charaoui-Boukerzaza (2009). These results are also generally reflected in our previously reported transcriptome data (Bellieny-Rabelo et al., 2019) where genes involved in glucose, D-galacturonate, citrate, valine, methionine, and aspartate uptake and metabolism were up-regulated in *Pb 1692* potato tuber infection (Bellieny-Rabelo et al., 2019). Malic acid is another major metabolite of plant cells, typically represented in root exudates as well as potato tuber extracts (Dobson et al., 2008; Uri et al., 2014; Feng et al., 2018, 2019). Yet, under these specific experimental conditions, we found that *Pb 1692* was unable to grow in malic acid and least responsive to sugars such as maltose. It must be noted that the concentration of a ligand can affect whether binding to the receptor occurs or not (Uri et al., 2014; Feng et al., 2018, 2019). Furthermore, *in vitro* concentrations and conditions may differ from those *in vivo*, thus it stands to reason that the outcome of our assays will have been greatly influenced by our specific experimental conditions.

The MCP mutant strains retained growth rates similar to those of the wild-type strain and were not affected in virulence. Therefore, we can conclude that these four MCP proteins do not contribute to the maceration of potato tubers. On the contrary, attachment assays showed that all the MCP mutants were significantly reduced in their ability to attach to potato leaf-surfaces compared to *Pb* 1692 wild-type. This would therefore suggest that MCPs play an important role in sensing chemo-attractants and subsequently initiating flagella-mediated swimming motility toward the host. Therefore, it can be argued that the ability of the *Pb* 1692 strain to sense and attach to potato leaves is a measure of its ecological fitness. SRP are ubiquitous by nature and can be found in different ecological niches such as soil, water and the host (Charkowski et al., 2012). Nonetheless, it has to be noted that survival outside the host for the SRP is only for short periods of time (Toth et al., 2021). Hence, the ability (conferred by MCPs as demonstrated here) to sense and move toward the host enables them to move from unfavorable niches to the host environment where survival is more likely (Van Gijsegem et al., 2021). As demonstrated in our results, these MCPs respond to xylose, aspartate, and citrate which are major metabolites found in various plant tissue types including potato tubers. However, it is important to take cognisance of the fact that a single chemoreceptor can bind several different ligands (Falke and Hazelbauer, 2001; Porter et al., 2011; Matilla and Krell, 2017). Thus, while these were the ligands that these mutant strains were least responsive to, it is important to note that our screening did not exhaust all potential ligands. It is therefore likely that these MCPs could bind many other ligands not tested in this study. For instance, in this study we identified a chemoreceptor that responds to Asp. A recent study by Cerna-Vargas et al. (2019) elegantly demonstrated that a *Pseudomonas syringae tomato* chemoreceptor, PsPto-PscA, responds to L-Asp, Glu and D-Asp (Cerna-Vargas et al., 2019). It would therefore be interesting to determine whether *Pb 1692* (MCP*_*asp*_*) also responds to other amino acids similar to those identified for PsPto-PscA.

Interestingly, two of the MCP mutant strains appear to be affected in their response to citrate. Protein alignment of these two proteins indicated that these have a 61 % similarity. Thus these two proteins appear to be paralogs and could be dependent on each other in sensing citrate ligands in *Pb 1692* strain. Possibly, effective chemotactic action toward citrate by *Pb 1692* could be synergistically performed by these two MCP paralogs or they could extend the response range of *Pb 1692* to citrate. This phenomenon was observed in *Pseudomonas putida* KT2440 where McpQ and McpS are paralogous chemoreceptors both involved in sensing citrate ligands (Martín-Mora et al., 2016). It is noteworthy that architectures of MCP_*cit2*_ and MCP_*cit1*_ present transmembrane regions in similar locations (Supplementary Figures 1, 2); furthermore, while the first protein remains with both HAMP-MCP domains, the latter conserves HMB-MCP, suggesting that the ancestor-gene architecture could be HMB-HAMP-MCP since there is no sequence-overlapping among the copies’ domains (Supplementary Figure 2). In addition, similar to *Salmonella typhimurium* TCP, the LBD of the two *Pb* MCP_*cit*_ is 4HB while the *P. petuda* McpQ forms a 4HMB fold (Martín-Mora et al., 2016). This demonstrates how diverse bacterial receptors that recognize citrate are and thus underscores the importance of citrate as a major component in plants.

In conclusion, this study has cataloged some of the chemo-attractants that *Pb 1692* responds to. Furthermore, mutation of four *Pb 1692* MCPs resulted in attenuated ability to adhere to their host implying that intact chemotaxis machinery in the *Pb 1692* strain is required for effective colonization during the early stages of infections. Our findings also show that the four MCPs of *Pb 1692* could be associated with chemo-attractants such as xylose, aspartate, and citrate.

## Materials and Methods

### Bacterial Strain and Growth Conditions

All bacterial strains and plasmids used in this study are indicated in Table 2. The *Pectobacterium brasiliense 1692* strains were regularly cultured in liquid Luria-Bertani (LB) broth or M9 media at 37°C with constant shaking (370 rpm) (Sambrook et al., 1989). Growth medium was supplemented with either 50 μg/ml kanamycin (Sigma-Aldrich) or 100 μg/ml ampicillin (Sigma-Aldrich) for mutant and complementation strains, respectively. When required, growth media was supplemented with amino acids and other carbon sources to a final concentration of 1 mM.

**TABLE 2:** Bacterial and plasmid strains were used in this study.

Bacterial strains/plasmid	Characteristic	Source
**Bacteria strains**		
*Pectobacterium brasiliense 1692*	Isolated from potato in Brazil, sequenced strain	
*Pb 1692*Δ*MCP*_*cit1*_	*Pb 1692* with a deletion in the gene encoding *AED-0003671*, Kan*^r^*	This study
*Pb 1692*Δ*MCP*_*cit2*_	*Pb 1692* with a deletion in the gene encoding *AED-0001492*, Kan^r^	This study
*Pb 1692*Δ*MCP*_*xyl*_	*Pb 1692* with a deletion in the gene encoding *AED-0000304*, Kan^r^	This study
*Pb 1692*Δ*MCP*_*asp*_	*Pb1692* with a deletion in the gene encoding *AED-0000744*, Kan^r^	This study
*Pb 1692*Δ*MCP_*cit1*_-pcit1*	*Pb 1692*Δ*MCP*_*cit1*_ with pJET1.2 bacterial cloning vector expressing the *AED-0003671* gene insert, Amp^r^	This study
*Pb 1692*Δ*MCP*_*cit2*_-p*cit2*	*Pb 1692*Δ*MCP*_*cit2*_ with pJET1.2 bacterial cloning vector expressing the *AED-0001492* gene insert, Amp^r^	This study
*Pb 1692*Δ*MCP*_*xyl*_-p*xyl*	*Pb 1692*Δ*MCP*_*xyl*_ with pJET1.2 bacterial cloning vector expressing the *AED-0000304* gene, Amp^r^	This study
*Pb 1692*Δ*MCP*_*asp*_-p*asp*	*Pb 1692*Δ*MCP*_*asp*_ with bacterial cloning vector expressing the *AED-0000744* gene, Amp^r^	This study
**Plasmids**		
pKD4	Plasmid containing a Kan^r^ cassette flanked by FTR sites	
pKD20	Plasmid expressing the lambda red genes	
pJET1.2/blunt	Cloning vector, Amp^r^	Thermo-Fisher

*Amp^*r*^ and Kan^*r*^ resistance to ampicilin and kanamycin, respectively.*

### *In silico* Identification, Alignment, and Domain Recognition of Methyl-Accepting Proteins in *Pb 1692*

Previously identified MCP gene homologs from *D. dandantii* (Rio-Alvarez et al., 2012) were used as bait to identify homologous genes in *Pb 1692*, using (BLASTN) in the ASAP database^2^. The search for the conserved domain in *Pb 1692* MCPs was performed using the Pfam identifier. Using HMMER v 3.0, the alignment of MCPs proteins was used to construct hidden Markov models (HMMs) to search and identify conserved domains. Amino acid alignment of two MCPs was performed using BLASTP (Altschul et al., 1990) and visual processing by Jalview (Waterhouse et al., 2009). Recognition of conserved domains in protein sequences was made by using HMMER3 package, and transmembrane regions prediction by Phobius; graphical representation of these results was made by IBS software.

### Construction of Mutants

The four methyl-accepting chemotaxis genes (*AED-0001492*, *AED-0003671*, *AED-0000304*, and *AED-0000744*) in the *Pb 1692* wild-type strain were inactivated using the lambda red recombinase one-step PCR inactivation method as previously described by Datsenko and colleagues (Datsenko and Wanner, 2000; Tanui et al., 2017; Supplementary Figure 3). Briefly, the open reading frames (ORF) of the targeted genes were replaced by a kanamycin resistance gene. Using kanamycin specific primers with upstream and downstream extended nucleotides, the kanamycin resistance gene was PCR-amplified from pKD4. Subsequently, upstream and downstream of the target gene were PCR amplified, excised, and purified using gel extraction kits following manufactures instruction (Zymo PCR purification kit). The three PCR products were fused using 5′ forward and 3′ reverse primers denoted with an asterisk^∗^ (Table 3). The fused products were gel purified and electroporated into electro-competent *Pb 1692* cells carrying pKD20 plasmid. All primers used in this study are listed in Table 3. Each PCR reaction contained 1 μl of DNA template, 12.5 μl of HiFi HotStart PCR Kit (KAPA Biosystems), 10 μl of nuclease-free H_2_O, and 0.75 μl (0.5 μM) of each forward and reverse primer making a reaction volume of 25 μl. The PCR conditions were 96°C for 3 min, 30 cycles of 98°C for 30 s, 60°C for 30 s, and 72°C for 2 min, followed by 72°C for 2 min. Detection of kanamycin insert was confirmed using test primers which bind up and downstream of the gene thus amplify the upstream, kanamycin, and downstream sequences of the gene (Table 3). The transformants were selected on an LB agar plate supplemented with 50 μg/ml^–1^ kanamycin (Table 2). The number of kanamycin insertion sites on *Pb 1692* was determined using Southern blot hybridization.

**TABLE 3:** Primers used in this study (*represents primers used for fusion).

Primer	5′-3′ nucleotides	Source
name		
	***Pb1692*Δ*MCP*_*cit1*_**	

TFcit1	Agcatggaagaactgacatcg	This study
TRcit1	Aaccatgttcgggttgttgtg	This study
F1cit1*	Agccaaatcagtactgaagcctc	This study
R1cit1	Cgaagcagctccagcctacacatgcattataactctccatgtataacg	This study
kFcit1	Cgttatacatggagagttataatgcatgtgtaggctggagctgcttcg	This study
kRcit1	gtgaagtatccgggcgtgaggcgttaaacatatgaatatcctccttagttcctattccgaag	This study
F2cit1	cttcggaataggaactaaggaggatattcatatgtttaacgcctcacgcccggatacttcac	This study
R2cit1*	Ttggttgcgtgatgcgtctgc	This study

	***Pb1692*Δ*MCP*_*cit2*_**	

TFcit2	Gattcacaccatgcagcacac	This study
TRcit2	Ttcgttcctgctctcatgacc	This study
F1cit2*	Acatttcaatctgcgtgtcgtc	This study
R1cit2	Gaagcagctccagcctacacagctaagaacatgacgtctctccgg	This study
KFcit2	Ccggagagacgtcatgttcttagctgtgtaggctggagctgcttc	This study
KRcit2	Cagggctgaaggatcgaacgttagcatatgaatatcctccttagttc	This study
F2cit2	Gaactaaggaggatattcatatgctaacgttcgatccttcagccctg	This study
R2cit2*	Cgcataacgattattcagagc	This study
TFcit2	Gattcacaccatgcagcacac	This study

	***Pb1692*Δ*MCP*_*asp*_**	

TFasp	Aaagaaggcgactggattgc	This study
TRasp	Accgagtaatgggcaacgtag	This study
F1asp*	Gtcttactgttacacggaacg	This study
R1asp	Cgaagcagctccagcctacacacttacgaaacataaattatccctg	This study
KFasp	Cagggataatttatgtttcgtaagtgtgtaggctggagctgcttcg	This study
KRasp	Gccatttaacgattagcgggcatcatatgaatatcctccttagttc	This study
F2asp	Gaactaaggaggatattcatatgatgcccgctaatcgttaaatggc	This study
R2asp*	Aggtatcgctgagcgaaagtg	This study

	***Pb1692*Δ*MCP*_*xyl*_**	

TFxyl	Gctctgcgcgatgcggatatc	This study
TRxyl	Ctatgcaggtcgtagacgcag	This study
F1xyl*	Ccagctccaacttcggtaacg	This study
R1xyl	Cgaagcagctccagcctacacagggatttcataggtgtgctc	This study
KFxyl	Gagcacacctatgaaatccctgtgtaggctggagctgcttcg	This study
KRxyl	Gtcttaatgcactaccttgataacagcgcatatgaatatcctccttagttc	This study
F2xyl	Gaactaaggaggatattcatatgcgctgttatcaaggtagtgcattaagac	This study
R2xyl*	Ttcatcggcatcgctatcttg	This study
TFxyl	Gctctgcgcgatgcggatatc	This study
	**Complementation primers**	

	***Pb1692* Δ*MCP*_*cit1*_-p*cit1***	

CFcit1F	Tggaccaccttctaacgttcg	This study
CRcit1R	Ttgataccgctatagggttcc	This study

	***Pb1692*Δ*MCP*_*cit2*_-p*cit2***	

CFcit2	Tcactaatcggtatacttcac	This study
CRcit2	Acgtcatatcagggctgaagg	This study

	***Pb1692*Δ*MCP*_*asp*_-p*asp***	

CFasp	Agataccagcggacatggcac	This study
CRasp	Ctcagatagggtctagtgttg	This study

	***Pb1692*Δ*MCP*_*xyl*_-p*xyl***	

CFxyl	Tgagcccttcttacctcttcac	This study
CRxyl	Aggacagcagatactgctgtc	This study

### Construction of the Complemented Mutant Strains

The complemented strains were generated by amplifying individual methyl-accepting genes *AED-0001492*, *AED-0003671*, *AED-0000304*, and *AED-0000744* with their cognate promoter region in *Pb1692* using primers CFcit2/CRcit2, CFcit1F/CRcit1R, CFxyl/CRxyl, and CFasp/CFasp set of primers, respectively (Table 3). The DNA fragments were cloned into the bacterial cloning vector pJET1.2 (Thermo-Fisher) to generate pJet*cit2*, pJet*cit1*, pJet*xyl*, and pJet*asp*, which were individually electroporated into the electrocompetent corresponding mutant strains to generate the complemented mutant strains *Pb1692*Δ*MCP*_*cit2*_-p*cit2*, *Pb1692*Δ*MCP*_*cit1*_-p*cit1*, *Pb1692*Δ*MCP*_*xyl*_-p*xyl*, and *Pb1692*Δ*MCP*_*asp*_-p*asp*. The transformants were selected on LB agar plate amended with 100 μg/ml^–1^Ampicilin (Table 2). All generated complements were further confirmed using PCR and DNA sequencing.

### Growth Curve Analysis

To determine what effect deletion of the different *Pb 1692* MCP genes has on the growth of the mutant strains when compared to the *Pb 1692* wild-type strain, a growth rate curve was performed. *Pb 1692* wild-type and the corresponding MCP mutant strains generated in this study were grown in LB broth at 37°C for 16 h with continuous agitation at 370 rpm. The optical density (OD) of the cultures were adjusted to an OD_600_ equivalent to 0.4 (OD_600_ = 0.4). Two milliliters of each *Pb 1692* strain was inoculated into 100 ml of LB broth and incubated at 37°C with constant agitation at 370 rpm. The optical density (OD_600_) of each culture was recorded every 1 h for 18 h. The experiment was performed in triplicates, and the mean values were used to calculate statistically significant differences between the wild-type and the MCP mutant strains using a one-way ANOVA. *P*-values less than 0.05 (*p* < 0.05) were considered to represent a statistically significant difference.

### Chemotaxis and Motility Assays on Agar Plates

Chemotaxis and motility tests were investigated on semi-solid 0.3 % agar minimal media medium. Each plate was individually supplemented with different chemo-attractant at the same concentration (1 mM). Optical densities of all strains were standardized (OD_600_ = 1). A 5 μl of bacterial suspension aliquot was pipetted at the center of each plate then incubated at 37°C for 24 h. Motility toward attractant between *Pb 1692* wild-type and the mutant strains was compared 24 h after incubation. Chemotaxis assays were generally performed no less than three independent occasions with at least three plates per assay.

### Attachment Assays

Attachment and entry of the *Pb 1692* wild-type, *Pb*1692Δ*MCP*_*cit1*_, *Pb*1692Δ*MCP*_*cit2*_, *Pb1692*Δ*MCP*_*asp*_, and *Pb1692*Δ*MCP*_*xyl*_ into potato leaves was investigated. To this end, *Pb 1692* cultures were prepared in M9 minimal media as previously described. Thereafter, potato leaves with a diameter of 3 cm were dipped into 10 ml of M9 minimal media inoculated with 10 μl of each strain equivalent to a density of 10^7^ CFU/ml. Minimal media was supplemented with different ligands to a final concentration of 1mM. Inoculated leaves were incubated at 28°C for 2 h without shaking. Thereafter, the leaves were removed washed with double distilled water and crushed by grinding in 1 ml of 10 mM MgSO_4_. Viable cell counts were determined by serial dilution and plating on LB medium. Unwounded potato leaves were used as the control experiment. Each experiment consisted of three biological and three technical repeats.

### Virulence Assays

Virulence assays were performed as described previously (Moleleki et al., 2017). Briefly, fresh and healthy susceptible potato tubers (cv Mondial) were socked in 10% sodium hypochlorite for 10 min, rinsed twice with double-distilled H_2_O, and air-dried. The optical density of all *Pb 1692* strains was adjusted to 1 (OD_600_ = 1). A 1 cm hole was made in sterilized potato tubers using sterilized tips and 10 μl of each strain was inoculated onto each hole. All *Pb 1692* strains (wild-type, mutants, and complemented strains) were inoculated in a single potato tuber, including 10 mM MgSO_4_ which served as a control. Inoculated potato tubers were then sealed with petroleum jelly and incubated in moist sterilized plastic boxes at 25°C for 72 h. Thereafter, the macerated tissue from the inoculated site was scooped and quantified. Three biological experiments with three technical repeats were performed.

### *In planta* Multiplication Analysis

To investigate the mutants’ multiplication ability during *in planta* infection, *Pb 1692* strains were inoculated into potato tubers as described in the virulence assays. For bacterial CFU/ml enumeration, 0.2 g of macerated tissue was resuspended in 1 ml of double-distilled H_2_O. Plant tissue was removed by centrifuging at 11904.464 *g* for 3 min, and the supernatant which contains bacteria were collected and serially diluted on plates containing appropriate antibiotics. The plates were incubated at 37°C for 24 h for CFU counts. Three biological experiments with three technical repeats were performed.

### Statistical Analysis

In this study, experiments were performed in triplicate and three independent times. Where applicable, a one-way Analysis of Variance (ANOVA) was performed to determine statistical significance and a *p*-value less than 0.05 (*p* < 0.05) was considered to be a statistically significant difference.

## Data Availability Statement

The original contributions presented in the study are included in the article/Supplementary Material, further inquiries can be directed to the corresponding author/s.

## Author Contributions

CKT and LNM contributed to the conception and design of the study. CKT and PKS performed the experiment, organized, and analyzed the data. CKT, PKS, and DB-R performed the bioinformatics and statistical analyses. CKT wrote the first draft of the manuscript. LNM provided resources and supervised this study. DYS generated all mutant strains and complementation plasmids. All authors contributed to the manuscript reviewed, edited, and approved the submitted version.

## Conflict of Interest

The authors declare that the research was conducted in the absence of any commercial or financial relationships that could be construed as a potential conflict of interest.

## Footnotes

1 http://asap.ahabs.wisc.edu/asap/home.php

2 http://asap.ahabs.wisc.edu/asap/home.php
