# Supplementary material for: Pectobacterium brasiliense 1692 Chemotactic Responses and the Role of Methyl-Accepting Chemotactic Proteins in Ecological Fitness
Source: Front Plant Sci. 2021 Apr 22;12:650894. doi: 10.3389/fpls.2021.650894 (PMC8100591; doi:10.3389/fpls.2021.650894)
Supplement: Supplementary file 1 [file Data_Sheet_1.pdf]

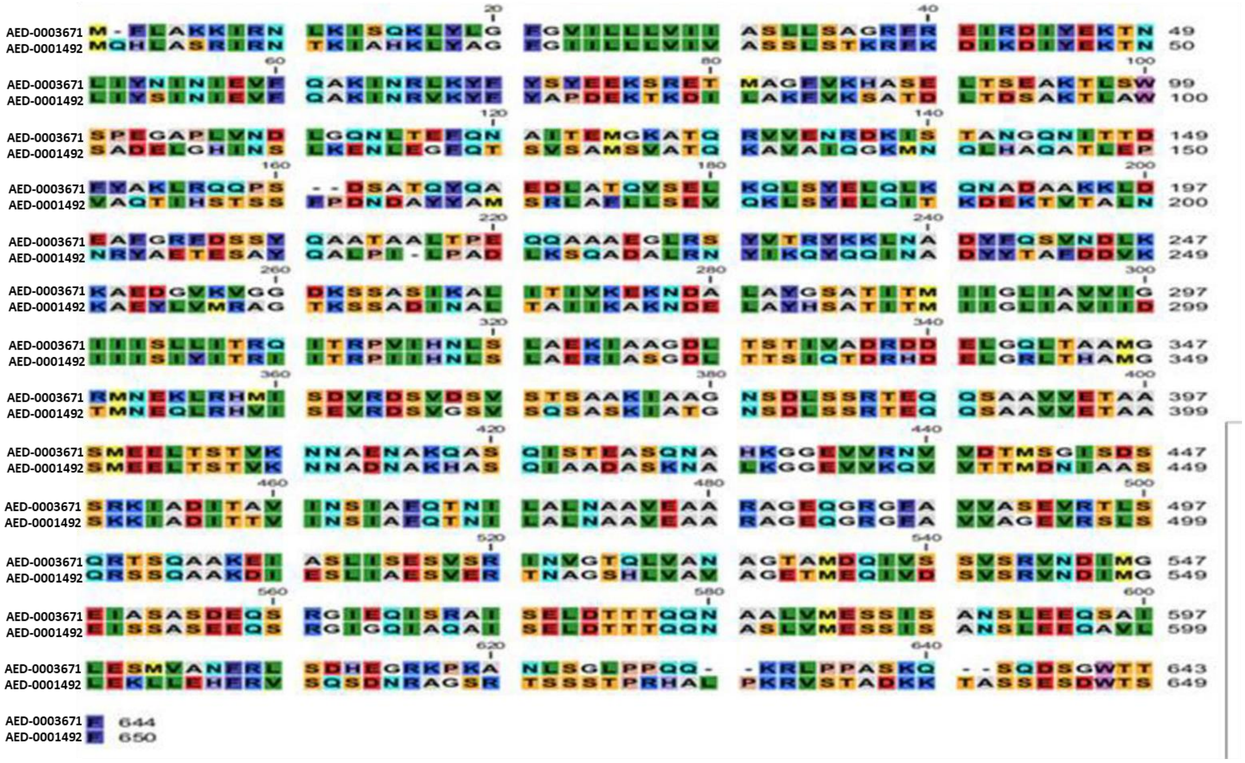

Fig S1. Alignment of the AED-0001492 and AED-0003671 proteins associated with *Pb 1692* methyl-accepting proteins (MCPs). The alignment shows 61 % similarity between AED-0001492 and AED-0003671 proteins.

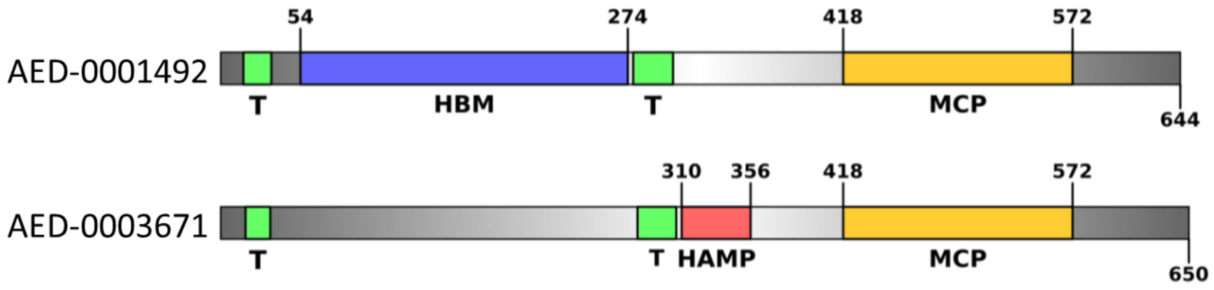

Fig S2. Schematic diagram showing AED-0001492 and AED-0003671 domains

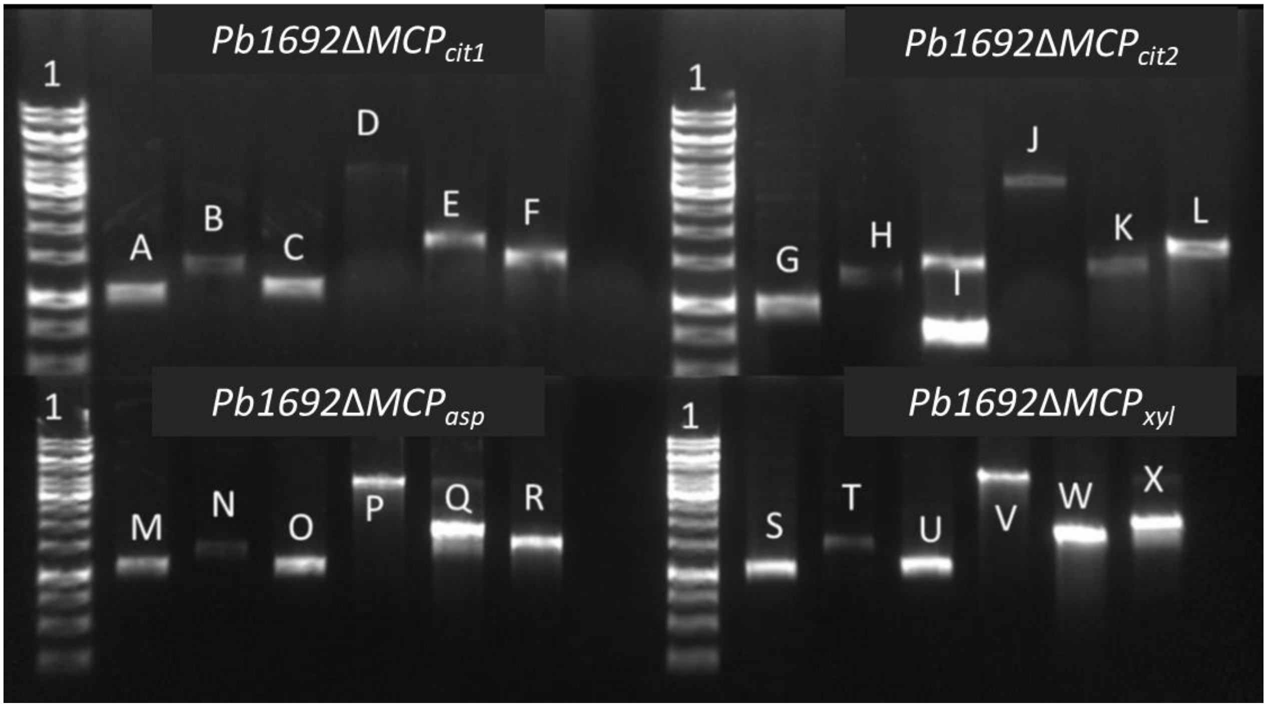

Fig S3. PCR amplicons used to generate and confirm mutant strains. Downstream amplicon A, G, M and S. kanamycin cassette amplicon B, H, N and T. The upstream PCR product included C, I, O and U. Fused downstream, kanamycin cassette and upstream D, J, P and V. PCR product confirming kanamycin insert, downstream forward primer and kanamycin internal reverse primer E, K, Q and W. PCR product confirming kanamycin cassette insert, upstream reverse primer and kanamycin internal forward primer F, L, R and X, 1 represents 1 kb ladder.

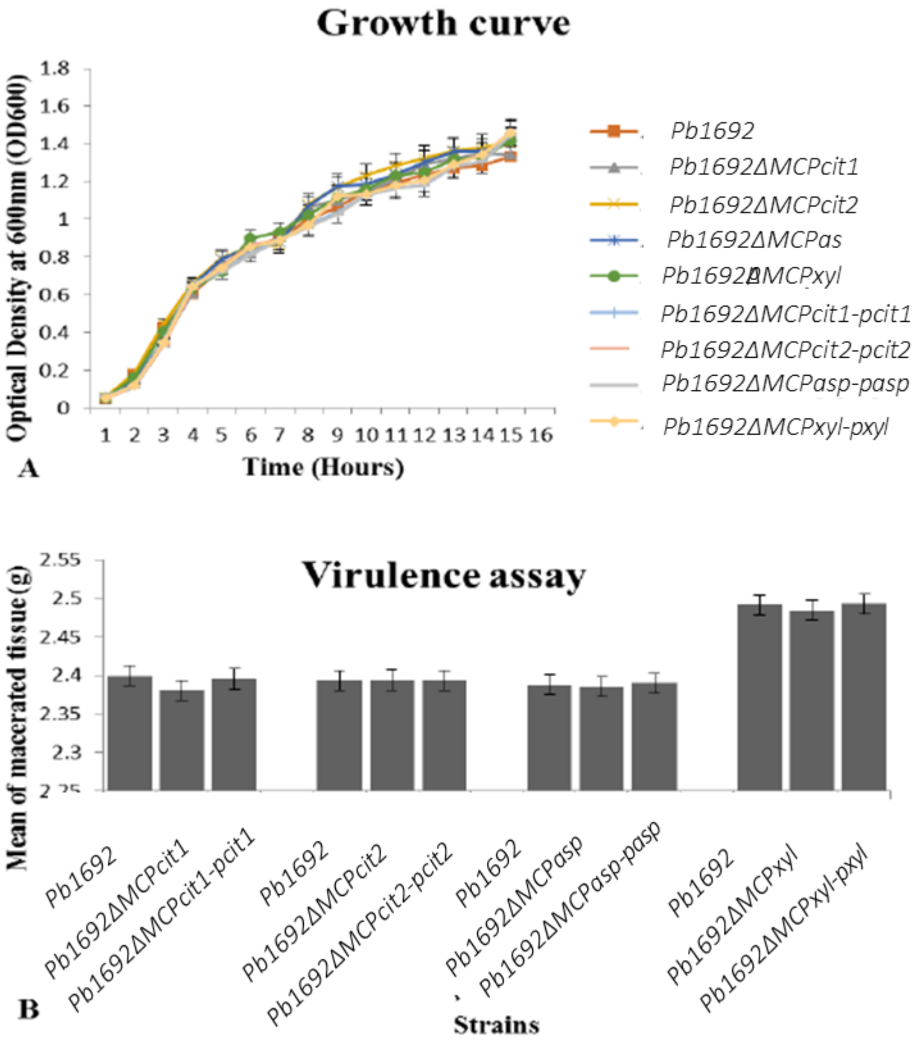

Fig S4. Mutation of MCPs has no effect on growth and virulence on potato tubers. A) *In vitro* growth of *Pb 1692* wild-type and their derivatives *Pb1692ΔMCPasp*, *Pb1692ΔMCPcit2*, *Pb1692ΔMCPcit1* and *Pb1692ΔMCPxyl* and their complement strains *Pb1692ΔMCPcit1*-p*cit1*, *Pb1692ΔMCPcit2*-p*cit2*, *Pb1692ΔMCPasp*-p*asp* and *Pb1692ΔMCPxyl*-p*xyl* in LB broth supplemented with appropriate antibiotics. B) Similarly, maceration on potato tubers following inoculation of the *Pb 1692* wild-type and mutant strains had no significant difference. Bars indicate standard error of the mean.
